# Supplementary material for: Rural-Urban differentials in prevalence, spectrum and determinants of Non-alcoholic Fatty Liver Disease in North Indian population
Source: PLoS One. 2022 Feb 10;17(2):e0263768. doi: 10.1371/journal.pone.0263768 (PMC8830644; doi:10.1371/journal.pone.0263768)
Supplement: S1 Table — (DOCX) [file pone.0263768.s001.docx]

**S1 Table. Crude prevalence (%) of NAFLD based on different parameters in urban Delhi and rural Ballabhgarh**

| Particular | Urban (N=828) | | | Rural (N=832) | | |
| --- | --- | --- | --- | --- | --- | --- |
|  | Men %(95%CI) | Women %(95%CI) | Total %(95%CI) | Men  %(95%CI) | Women %(95%CI) | Total  %(95%CI) |
| NAFLD on USG | 64.5 (59.7,69.1) | 69.3 (64.8,73.4) | 67  (63.7,70.2) | 59.7 (54.2,65.1) | 60.7 (56.4,64.8) | 60.3  (57,63.6) |
| NAFLD (on USG) with ALT≥40 IU/L | 31.9 (27.4,36.7) | 13.3 (10.4,16.8) | 22.1 (19.4,25.1) | 30.4 (25.5,35.7) | 11.8 (9.2,14.8) | 18.8 (16.2,21.6) |
| NAFLD on CAP scores | 59.9  (55,64.7) | 52.8  (48,57.4) | 56.2 (52.8,59.5) | 36.7 (31.6,42.2) | 30.8  (27,34.9) | 33.1 (29.9,36.3) |
| Any Fibrosis | 19.9  (16.2,24.2) | 15.8  (12.7,19.6) | 17.8 (15.3,20.5) | 7.0 (4.7,10.5) | 5.0  (3.4,7.3) | 5.8  (4.4,7.6) |
| Cirrhosis | 3.06  (1.74,5.32) | 2.98  (1.74,5.07) | 3.02 (2.05,4.43) | 0.96 (0.31,2.94) | 0.19 (0.03,1.36) | 0.48 (0.18,1.28) |

#for all definitions see Box 1
