# Supplementary material for: Rural-Urban differentials in prevalence, spectrum and determinants of Non-alcoholic Fatty Liver Disease in North Indian population
Source: PLoS One. 2022 Feb 10;17(2):e0263768. doi: 10.1371/journal.pone.0263768 (PMC8830644; doi:10.1371/journal.pone.0263768)
Supplement: S2 Table — (DOCX) [file pone.0263768.s002.docx]

**S2 Table.** **Univariate and Multivariate analysis of factors associated with NAFLD defined on ultrasound in the combined dataset**

|  | **Univariate** | **Multivariate** |
| --- | --- | --- |
|  | **OR (95%CI)** | **OR (95%CI)** |
| **Age 45-60 years** | 1.06 (0.87-1.30) | 0.85 (0.67-1.08) |
| **Female Gender** | 1.10 (0.90-1.35) | 0.92 (0.72-1.19) |
| **Place of residence-urban** | 1.34 (1.09-1.63) | 1.04 (0.82-1.33) |
| **Diabetes** | 3.46 (2.59-4.63) | 2.08 (1.49-2.91) |
| **Hypertension** | 1.63 (1.33-2.01) | 1.05 (0.81-1.37) |
| **Overweight** | 2.24 (1.63-3.09) | 1.59 (1.12-2.27) |
| **Central Obesity** | 5.10 (4.05-6.42) | 2.03 (1.44-2.85) |
| **Total cholesterol**  **>=200 mg/dl** | 1.48 (1.19-1.84) | 1.19 (0.91-1.56) |
| **Triglyceride**  **≥150 mg/dl** | 1.99 (1.61-2.46) | 1.30 (1.01-1.67) |
| **Reduced HDL** | 1.31 (1.07-1.60) | 1.03 (0.80-1.33) |
| **Homa IR** | 4.12 (3.33-5.09) | 1.88 (1.45-2.43) |

Diabetes: Fasting plasma glucose≥126 mg/dl and/ or HbA1c ≥6.5% and/or reported being on anti-diabetes medication; Hypertension: Systolic blood pressure ≥ 140 mmHg or diastolic blood pressure ≥90 mmHg or on antihypertensive drug for hypertension; Central obesity: waist circumference ≥90 cm for Asian men or ≥80 cm for Asian women; Reduced HDLc: HDL cholesterol<40 mg/dl in men or <50 mg/dl in women.
