# Supplementary material for: Rural-Urban differentials in prevalence, spectrum and determinants of Non-alcoholic Fatty Liver Disease in North Indian population
Source: PLoS One. 2022 Feb 10;17(2):e0263768. doi: 10.1371/journal.pone.0263768 (PMC8830644; doi:10.1371/journal.pone.0263768)

Annexure – 1

Screening form

Prevalence of Non-Alcoholic Fatty Liver Disease (NAFLD) and its association with cardio-metabolic disease risk factors in North India

**I am __(name)_______ I have come from (institution name) …….. (Introduction)**		
**Urban or Rural (Radio Button)**		
**SCREENING ID**	**Interviewer ID:**	
**Date of Interview:- (Tab generated)**	**Time of interview: (tab generated)**	
1. Name of the participant :		
2. Gender	1=Male<br>2=Female<br>3-Trans-gender	☐
3. Is the participant available ?	1=Yes<br>2= No, shifted, new address available<br>3= No, shifted not traceable<br>4= Participant deceased<br>5- others	☐<br>If "2", please go to the 3a<br>If "5", specify<br>______________
3a If changed, pls specify the address		
4. Name and Phone number		
5. Does the participant give verbal consent to be interviewed? If NO, what is the reason for refusal	1= Yes<br>2= No	☐<br>If 1, pls go to Q7
6. If refused, reason for refusal		
7. Is participant age between 30 and 60 years?	1=Yes<br>2=No<br>If 'NO' don't proceed further	☐
8. Is participant bed-ridden?	1=Yes<br>2=No<br>If YES, don't proceed further	☐

9. Do you suffer from any of the following liver disease? If ‘ YES’ for any of the following questions (request documents if available) , don’t proceed further		
a. Chronic liver disease/liver failure	1=Yes<br>2=No	☐
b. Cirrhosis	1=Yes<br>2=No	☐
c. Hepatocellular carcinoma	1=Yes<br>2=No	☐
10. Are you taking supplements for body building?<br>If YES, don’t proceed further	1=Yes<br>2=No	☐
11. Have you ever suffered from cancer or took any medication for cancer?<br>If YES, don’t proceed further	1=Yes<br>2=No	☐
12. A. Have you ever consumed alcohol?<br>If no for men, recruit participant after IC procedure<br>If no for women , go to question 13	1=Yes<br>2=No	☐
b. If yes, have you consumed alcohol in last one year?<br>If no for men, recruit participant after IC procedure<br>If no for women , go to question 13	1=Yes<br>2=No	☐
d. How often do you have a drink containing alcohol?<br>If ‘Less than once a week’ for men, recruit participant after IC procedure<br>If ‘Less than once a week’ for women , go to question 13	1= Once or more in a week<br>2= Less than once a week	☐
(Ask only for women)		
13. Is the participant pregnant? (only if the participant is woman)	1=Yes<br>2=No<br>3= Don’ know<br>If YES and Don’t Know, don’t proceed further	☐
14. Are you taking oral contraceptive pills or hormonal replacement therapy<br>If yes, don’t proceed further	1=Yes<br>2=No	☐

MAIN QUESTIONNAIRE

**Urban or Rural (Radio Button)**	
**SCREENING ID**	Interviewer ID:-
HOUSEHOLD ID	
Date of Interview:- **(Tab generated)**	Time of interview: **(tab generated)**
**Instruction to the interviewer: HAS THE PARTICIPANT SIGNED THE INFORMED CONSENT? DO NOT PROCEED UNTIL THE CONSENT FORM HAS BEEN SIGNED.**	
Consent	Yes/NO
If Consent Yes,<br><br>Scan next available barcode for STUDY ID (THIS HAS TO BE STUCK IN THE MASTER LIST NOTE DETAILS LIKE NAME SEX AND ADDRESS ETC.	

**NAFLD ID :**	Screening ID :
HOUSEHOLD ID	Interviewer ID :-
Date of Interview:-	Time of interview:
**CONTACT DETAILS - IN BLOCK LETTERS**	
1. Name of the participant :-	
2. Father's name	
3. Mother's name	
4. Spouse's name (*ask if married*)	
5. Address detail-<br><br>Postal code :-	
6. Telephone no-<br>Mobile 1:-<br>Mobile 2:-<br>Residence :-<br>Work :-	

7. Email Id (if any) :-		
8. Does the partiicpant has adhaar card?	Yes 1<br>No 2<br>Don't know/refused 3	
8a. If yes, please write the adhaar card Number	☐☐☐☐☐☐☐☐☐☐☐☐	
**SECTION-1: DEMOGRAPHIC AND SOCIO- ECONOMIC DETAILS**		
**PART-1A: DEMOGRAPHIC DETAILS**		
1.1 Age *(In completed years)*	☐☐	
1.2 Date of birth (if available)	☐☐ ☐☐ ☐☐<br>DD MM YY	
1.3 How many members are in your household most of the time?	Age >= 10 years ☐ Age <10 years ☐	
1.4 What is your marital status ?	1= Single<br>2=Married<br>3= Widow/Widower<br>4= Separated/Divorced<br>5=Other	☐<br>**If others (option 5), then specify**<br>------------------------------------
**PART-1B: SOCIO-ECONOMIC DETAILS**		
1.5 Number of years of formal education*<br>***The total number of years the participant spent in any educational institution (schools, colleges, religious schools etc.)***		☐☐
1.6 Educational status (highest attained degree)<br>** A person who can both read and write with understanding in any language without any formal education or passed any minimum educational standard.*<br>*** A person, who can neither read nor write*	1= Professional degree/post graduate<br>2= Graduate (B.A/B.Sc/B.Com/Diploma)<br>3= Secondary School /Intermediary (ITI course ,class XII/X or Intermediate)<br>4= High school (class V to IX)	☐<br>**If others (option 8), then specify**<br>------------------------------------

or can only read but cannot write in any language	5= Primary School (up to Class IV)<br>6=*Literate, no formal education<br>7=**Illiterate<br>8=Other	
1.7 What is your employment status?<br><br>**If yes go to Q1.8 otherwise skip to Q1.9**	1=Employed<br>2=Student<br>3-Housemaker<br>4=Retired<br>5=Un-employed<br>6=Other	**If others (option 6), then specify**<br><br>------------------------------------
1.8 If ***"Employed"**, what* is your current occupation?		**Please mention**<br>------------------------------------

Coding list for employment (for 1.8)

Professional, big business ,landlord (> 10 acre) , university teacher, class 1IAS/services officer, lawyer	1
Trained, clerical, medium business owner, middle level farmer (2-10 acre) , teacher, maintenance (in-charge), personnel manager	2
Skilled manual laborer, small business owner, small farmer (<1 acre)	3
Semi-skilled manual laborer, marginal land owner, rickshaw driver, army jawan, carpenter, fitter	4
Unskilled manual laborer, landless laborer	5

1.9 What is your total average household income per month (INR)?	1= <3000<br>2= 3000-10,000<br>3= 10,001-20,000<br>4= 20,001-30,000<br>5= 30,001-40,000<br>6= 40,001-50,000<br>7= >50,000<br>8= Refused<br>9= Don't know	
1.10 Do you have a separate room for cooking (Kitchen)?	1=Yes<br>2=No	
1.11 What is the fuel used for cooking?<br><br>**[If more than one source is used then note the source that is most commonly used]**	1= Coal/charcoal/kerosene<br>2= Induction/Electricity/gas (LPG)/solar/CNG(IGL)<br>3= Wood/dung<br>4= Others	**If others (option 4), then specify**<br><br>------------------------------------
1.12 What is the source of drinking water used at home?<br><br>**[If more than one source is used then note**	1= Public source<br>2= Private source(Shared)<br>3= Private source(Own)<br>4= Bottled water	**If others (option 6), then specify**

**the source that is most commonly used]**	5= Purified tap water<br>6= Others	------------------------------------
1.13 What is the toilet facility you use?	1= Public toilet<br>2= Shared toilet<br>3= Own flush toilet<br>4= Others	**If others (option 4), then specify**<br>------------------------------------
1.14 Which of the following do you own?<br>***[Yes=1;No=2]***	Television<br>Refrigerator<br>Washing machine<br>Microwave/OTG<br>Mixer-grinder<br>Smart /mobile phone<br>DVD player<br>Computer/Laptop<br>Internet connection<br>Car<br>Motor Cycle/ Scooter<br>Bicycle<br>Tractor<br>Thresher	
**SECTION 2 :- TOBACCO, PHYSICAL ACTIVITY , and DIET HABITS**		
**PART 2A:- TOBACCO USE**		
2.1 Have you **EVER** used tobacco in any form (smoking, chewing, snuff, etc)?	Yes 1<br>No 2	**If "2" go to part 2B**
2.2 In what forms have you consumed tobacco?<br>**[Yes=1 ; No=2]**	In a smoking form<br>In a chewed form<br>In any other form (snuff, toothpaste etc.)	

2.3 Do you currently* consume tobacco?<br>**[Yes=1; No=2]**<br>**Currently refers to within past 6 months*	Yes 1<br>No 2		**If "2" go to part 2B**	
2.4 In which form?<br>**[Daily =1; 1-6 days a week =2; Less than once a week =3]**		**Yes =1 , No =2**	**If yes, how often**	**If yes, start year (first time)**
	Smoking form			
	Chewed form			
	Any other form (snuff, toothpaste etc.)			

**PART 2B:- ALCOHOL USE**		
2.5 Have you **EVER** used alcohol?	Yes 1<br>No 2	**"2" go to PART 2C**
2.6 How often do you use alcoholic beverages?	Consuming alcohol more than once a week **1**<br>Consuming alcohol Less than once a week **2**<br>Used alcohol in the past (stopped more than 6months ago) **3**<br>Recently stopped alcohol (less Than 6 months ago) **4**	

2.7 History of alcohol use for both present and past users

Type of alcohol used	Have you ever consumed following items	Duration of use [For how long]			Frequency of use<br><br>(Fill any one column)				Quantity/ occasion ** (in ml)	If stopped, since how long?<br><br>If Q 2.6 filled with Option "3" & "4" this Q should be filled	
	Yes=1; No=2	Year	Month	Day	Per day	Per week	Per month	Per year	ml	Year	Month
**A)** Local spirits eg. Desi arrack, toddy etc							Since				
**B)** Spirits eg. Whisky, rum, brandy, gin, vodka											
**C)**Beer											
**D)**Wine											

Conversion: *Please use local measures in calculating the total consumption (in ml per occasion)

For A & B: 1 small peg=30ml;1 large peg=60ml;1 extra-large peg =90ml; 1 quarter =180ml; a half bottle =375 ml; full bottle=750ml

For C: 1glass of beer =approx.325ml; Beer Can= 500ml; Bottle of Beer= 650 ml

For D: 1glass of wine=100ml

PART 2C :- PHYSICAL ACTIVITY (Global Physical activity Questionnaire)

Next, I am going to ask you about the time you spend doing different types of physical activity in a typical week. Please answer these questions even if you do not consider yourself to be a physically active person.

Think first about the time you spend doing work. Think of work as the things that you have to do such as paid or unpaid work, study/training, household chores, harvesting food/crops, fishing, seeking employment. In answering the following questions 'vigorous-intensity activities' are activities that require hard physical effort and cause large increases in breathing or heart rate, 'moderate-intensity activities' are activities that require moderate physical effort and cause small increases in breathing or heart rate.

**Questions**			**Response**
2C-I: - ACTIVITY AT WORK			
2.8	Does your work involve **vigorous-intensity activity** that causes large increases in breathing or heart rate like *[carrying or lifting heavy loads, digging or construction work*] for at least 10 minutes continuously?<br><br>Activities are regarded as vigorous intensity if they cause as large increase in breathing and/or heart rate<br><br>*[Sawing hardwood, forestry (cutting, chopping, carrying wood, ploughing, cutting crops (sugarcane), digging, grinding (with pestle),laboring (shoveling sand, loading furniture(stoves, fridge), instructing sports aerobics, cycle rickshaw driving]*	Yes 1<br>No 2	☐<br><br>**If "2", go to Q.2.11**

	(USE SHOWCARD)<br><br>***Think only about those physical activities that you do for <u>at least 10 minutes</u> at a time.***		
2.9	In a typical week, on how many days do you do vigorous-intensity activities as part of your work?<br>"Typical week" means a week when a person is doing vigorous intensity activities and not an average over a period.<br>Valid response ranges from 1-7.	No. of days	☐
2.10	How much time do you spend doing vigorous-intensity activities at work on a typical day?<br>Think of one day you can recall easily. Consider only those activities undertaken continuously for 10 minutes or more.<br>Probe very high response (over 4 hours) to verify	Hours : Minutes	☐☐ ☐☐<br>Hours : Minutes
2.11	Does your work involve moderate-intensity activity that causes small increases in breathing or heart rate such as brisk walking [or carrying light loads] for at least 10 minutes continuously?<br><br>Activities are regarded as moderate intensity if they cause as large increase in breathing and/or heart rate<br><br>[washing (bating and brushing carpets, wringing clothes (by hand),gardening, digging dry soil (with spade), weaving, woodwork (chiseling, sawing, softwood), mixing cement (with shovel), laboring (pushing loaded wheelbarrow, operating jackhammer, walking with load on head, drawing water, tending animals]<br>(USE SHOWCARD)<br><br>Do not include walking. Again, think about only those physical activities that you did for at least 10 minutes at a time.	Yes 1<br>No 2	☐<br><br>If "2", go to Q.2.14
2.12	In a typical week, on how many days do you do moderate-intensity activities as part of your work?<br>Valid responses range from 1-7	No. of days	☐
2.13	How much time do you spend doing moderate-intensity activities at work on a typical day?<br><br>Think of one day you can recall easily. Consider only those activities undertaken continuously for 10 minutes or more.<br>Probe very high responses (over 4 hrs) to verify	Hours : Minutes	☐☐ ☐☐<br>Hours : Minutes
***2C-II:* - Travel to and from places**			
The next questions exclude the physical activities at work that you have already mentioned.<br>Now I would like to ask you about the usual way you travel to and from places. For example: to work, for shopping, to market, to place of worship.			

The introductory statement to the following questions on transport-related physical activity is very important. It asks and helps the participant to now think about how they travel around getting from place-to-place. This statement should not be omitted.			
2.14	Do you walk or use a bicycle (*pedal cycle*) for at least 10 minutes continuously to get to and from places?	Yes 1<br>No 2	☐<br>**If "2", go to Q.2.17**
2.15	In a typical week, on how many days do you walk or bicycle for at least 10 minutes continuously to get to and from places?<br>Valid responses range from 1-7	No. of days	☐
2.16	How much time do you spend walking or bicycling for travel on a **typical day**?<br><br>Think of one day you can recall easily. Consider the total amount of time walking or bicycling for trips of 10 minutes or more.<br>Probe very high responses (over 4 hrs) to verify.	Hours : Minutes	☐☐ ☐☐<br>Hours : Minutes
***2C-III:* - Recreational activities**			
The next questions exclude the work and transport activities that you have already mentioned.<br>Now I would like to ask you about sports, fitness and recreational activities (leisure).			
2.17	Do you do any vigorous-intensity sports, fitness or recreational (*leisure*) activities that cause large increases in breathing or heart rate like [*running or football]* for at least 10 minutes continuously?<br>Activities are regarded as vigorous intensity if they cause a large increase in breathing and/or heart rate.<br><br>*[Badminton, tennis, high-impact aerobics, aqua aerobic, fast swimming]*<br>***(USE SHOWCARD)***	Yes 1<br>No 2	☐<br>**If "2", go to Q.2.20**
2.18	In a typical week, on how many days do you do vigorous-intensity sports, fitness or recreational (*leisure*) activities?<br>Valid responses range from 1-7	No. of days	☐
2.19	How much time do you spend doing vigorous-intensity sports, fitness or recreational activities on a typical day?<br><br>Think of one day you can recall easily. Consider the total amount of time doing vigorous recreational activities for periods of 10 minutes or more.<br>Probe very high responses (over 4 hrs).	Hours : Minutes	☐☐ ☐☐<br>Hours : Minutes

2.20	Do you do any moderate-intensity sports, fitness or recreational *(leisure)* activities that causes a small increase in breathing or heart rate such as brisk walking,(*cycling, swimming, volleyball*)for at least 10 minutes continuously?<br><br>Activities are regarded as moderate intensity if they cause a small increase in breathing and/or heart rate.<br><br>*[Cycling, jogging, dancing, horse-riding, yoga, low-impact aerobics, cricket]*<br>***(USE SHOWCARD)***	Yes 1<br>No 2	**If "2", go to Q.2.23**
2.21	In a typical week, on how many days do you do moderate-intensity sports, fitness or recreational (*leisure*) activities?<br>Valid responses range from 1-7	No. of days	
2.22	How much time do you spend doing moderate-intensity sports, fitness or recreational (*leisure*) activities on a **typical day**?<br><br>Think of one day you can recall easily. Consider the total amount of time doing moderate recreational activities for periods of 10 minutes or more.<br>Probe very high responses (over 4 hrs).	Hours : Minutes	Hours : Minutes
2C-IV: **- Sedentary behavior**			
The following question is about sitting or reclining at work, at home, getting to and from places, or with friends including time spent [sitting at a desk, sitting with friends, travelling in car, bus, train, reading, playing cards or watching television], but **do not include time spent sleeping**.<br><br>***(USE SHOWCARD)***			
2.23	How much time do you usually spend sitting or reclining on a **typical day**?	Hours : Minutes	Hours : Minutes

***2C-V:* - Yoga practice question**					
Now I will ask you about performing yoga					
2.24	Do you practice **Yoga (which includes activities like asana, pranayam or mediation)**? Record the appropriate response		Yes 1<br>No. 2	☐<br>**If No, go to part 2D**	
2.25	If yes, how many days in a typical week do you perform the following (complete one row)<br>Valid response for number days 1-7	Yes= 1<br>No=2	Number of days in the week	On a usual /typical day that you do yoga, how much time do you spend doing yoga related activities, in case you do the activities in more than one session in a day please add all sessions done in a typical day	
				Hours	Minutes
2.25a	Asana		☐	☐☐	☐☐
2.25b	Pranayam		☐	☐☐	☐☐
2.25c	Meditation		☐	☐☐	☐☐

**PART 2D** :- DIET			
2.26 Are you a vegetarian?	1- Yes<br>2- No		☐
2.27 Do you take eggs?	1- Yes<br>2- No		☐
2.28 Usually, on an average which meals per week have you missed?	Meals	Yes=1, No=2	Number of days
	1. Breakfast		
	2. Lunch		
	3. Dinner		

2.29 Usually, on an average, how frequently do you use OIL left AFTER DEEP-FRYING and for what purpose like cooking vegetables/daal / parantha/deep frying	Never 1<br>Less than once a month 2<br>Once in a month 3<br>Twice in a month 4<br>2-3 times/week 5<br>Daily 6<br>Don't know 7<br>Other 8	☐<br>____________________<br>____________________

2.30 Usually, which oil you use for cooking and how much in a month?	Name of the oil	*[Yes=1; No=2]*	Brand name	Monthly consumption (in ml)
	Mustard oil	☐		☐
	Sunflower oil	☐		☐
	Soyabean oil	☐		☐
	Groundnut oil	☐		☐
	Ricebran oil	☐		☐
	Palm oil	☐		☐
	Sesame/til oil	☐		☐
	Cocount oil	☐		☐
	Olive oil	☐		☐
	Others specify<br>----------------------	☐		☐

2.31 Usually which fat you use for cooking and how much in a month? (Like (Butter, Ghee, Vanaspati))	Name of the Fat	*[Yes=1; No=2]*	Brand name	Monthly consumption (in gram)
	Butter	☐		☐
	Ghee	☐		☐
	Vanaspati	☐		☐
	Others specify<br>-----------------------	☐		☐
	Others specify			

2.32 Usually what type of milk do you consume?	None 1<br>Skimmed milk 2<br>Double toned 3<br>Toned/cow's milk 4<br>Full cream/buffalo's milk 5<br>Don't know 6<br>Others specify 7	☐
2.33 How often is the meat you eat usually trimmed of fat?<br>[Don't ask this *question to vegetarians]*	Usually **(and who do not eat meat) 1**<br>Sometimes 2<br>Rarely or never 3<br>Do not know 4	☐

2.34 In the **PAST ONE YEAR**, how often have you consumed foods from the following food groups? **[Write the frequency of consumption in the appropriate column]**

	**Food items**	**Daily-1; Weekly-2; Monthly-3, Never or less than once a month-4**	**Frequency**		**Approx. amount eaten at one time (refer to show cards)**	
			**No. of times per month/week**	**No. of times/ day**		
1.	Meats [lamb, mutton, goat, veal, rabbit, beef, pork; their curries]					Pcs
2.	Poultry [chicken, turkey, duck, pheasant, quail; their curries]					Pcs
3.	Organ meats [liver, kidney, brain, spleen, heart and sausages nihari, paya]					Bowl
4.	Fish [fresh-water and sea-water fish; preserved fish such as salted fish, canned fish, dried fish]					Pcs
5.	Shell fish and crustaceans [crab, squid, prawns, molluscs]					Pcs
6.	Eggs [Includes preserved eggs, duck eggs]					Pcs

	Food items	Daily-1; Weekly-2; Monthly-3, Never or less than once a month-4	Frequency		Approx. amount eaten at one time (refer to show cards)	
			No. of times per month/week	No. of times/ day		
7.	Cooked green leafy vegetables (spinach, fenugreek, bathua, mustard, turnip greens, amaranth etc.)					Bowl
8.	Cooked other vegetables [beans, cauliflower, brinjal, ladies finger, pumpkin, bottle/bitter gourd, carrot, radish, onion, beetroot]					Bowl
9.	Cooked vegetables: roots and tubers[Potatoes, sweet potato, colocasia, Yam]					Bowl
10.	Cooked vegetables: add extra salt					Spoon
11.	Uncooked raw vegetables : salads with salt					Bowl
12.	Uncooked raw vegetables : salads without salt					Bowl
13.	Fruits (1) banana, cheeko/sapota, mango, grapes : with salt					Pcs
14.	Fruits (1) banana, cheeko/sapota, mango, grapes: without salt					Pcs
15.	Fruits (2) All other fruits: with salt					Bowl
16.	Fruits (2) All other fruits: without salt					Bowl
17.	Rice (Boiled rice, fried rice, briyani, pulav), semolina, sago, pasta					Bowl
18.	White bread, idli, dosa					Pcs
19.	Whole wheat roti/multi grain roti, brown bread,					Pcs
20. .	whole grain porridge, pearl millet, barley, ragi, oats with milk					Bowl
21.	whole grain porridge, pearl millet, barley, ragi, oats without milk					Bowl
22.	Add Salt in cooking rice/kneading dough					Spoon
23.	Legumes and pulses [includes all dals, black & white chana, rajma, lobia etc.)					Bowl
24.	Legumes and pulses: add extra salt					Spoon
	**Food items**	**Daily-1; Weekly-2; Monthly-3, Never or less**	**Frequency**		**Approx. amount eaten at one time (refer to show cards)**	

		than once a month-4				
			No. of times per month/week	No. of times/ day		
25.	Add extra Ghee/butter (Daal/vegetable/chapatti/Parantha)					spoon
26.	Milk & milk based drinks (all milk shakes)					Glass
27.	Milk products [yogurt, paneer, curd, raita, lassi]					Bowl
28.	Milk based desserts [custard, khoya, firni, kheer, milk puddings,Rabri, Milkcake]					Bowl
29.	Deep fried foods1 [chicken nuggets, onion rings, pakoras, namakparay, French fries, etc..]					Pcs
30.	Deep fried foods 2 [samosas, egg rolls, kachori, bread pakore, cutlets, poori, patties, Bathura]					Pcs
31.	Desserts1 [chocolate, tarts , cakes, pies, ice-creams & pastries]					Pcs
32.	Desserts2 [burfi,ladoo, jalebi, gulabjamum, rasgullah, rasmalai, Malpua]					Pcs
33.	Carbonated beverages					Glass
34.	Fresh fruit juices					Glass
35.	Fruit juices [Frozen (tetra-packed); Sherbets, Frooti, Maza etc.]					Glass
36.	Nuts [peanuts, almonds, cashews, walnuts etc.]					Pcs
37.	Sugar added to tea/coffee/cereals etc					spoon
38.	Tea/coffee [tea without milk and sugar and any other tea]					Cup/mug
39.	Tea/coffee with milk and sugar					Cup/mug
40.	Pickles [achar, pickled vegetables, roasted papad)					Pcs
41.	Sauces and chutneys					spoon
42.	Miscellaneous foods [biscuit, rusk, phen]					PCs

SECTION– 3:MEDICAL HISTORY (CARDIO METABOLIC DISEASES AND THEIR RISK FACTORS)

PART 3A: DISEASE SPECIFIC QUESTIONS

***3A-I:* HYPERTENSION (High Blood Pressure)/DIABETES (High Blood Sugar)/ HYPERLIPIDEMIA (High Blood Cholesterol)**

**Exclude pregnancy induced Hypertension and High Blood Sugar*

	Hypertension	Diabetes (High Blood Sugar)*	Hyperlipidemia (High Blood Cholesterol)

	(High Blood Pressure)*		
3.1 Have you **EVER** been told by a doctor that you have any of the following diseases? (except when you were pregnant)<br><br>***[Yes =1; No =2; Don't know=3]***	☐	☐	☐
Fill this section if the answer for high blood pressure/ high blood sugar/high blood cholesterol is "YES" in PART 3A-*I, *Q.3.1. *If the answer is **'YES'** to any of the choices in Q. 3.1, then go to Q.3.2. **'OTHERWISE'** skip the entire section and go to 3A*-II			
3.2 **When were you diagnosed?**	MM: ☐☐ YYYY ☐☐☐☐	MM: ☐☐ YYYY ☐☐☐☐	MM: ☐☐ YYYY ☐☐☐☐
3.3 What treatment are you taking for it currently? **[Yes=1; No=2]**			
a) Prescribed dietary modification	☐	☐	☐
b) Prescribed physical exercise	☐	☐	☐
c) Traditional medicine/Therapy*	☐	☐	☐
d) Allopathic drugs (English/modern)	☐	☐	☐
e) None	☐	☐	☐
***Traditional medicine/therapy include Yoga , Ayurveda, Unani, Homeopathy, Tibetan, Naturopathy, Meditation**			

**3A-II: HEART DISEASE**		
3.4 Have you **EVER** been told by a doctor that you have heart disease? **[Yes=1 ; No=2; *Don't know=3*]**	☐ **If "2" or "3" skip to *3A-III***	
3.5 What did the doctor say it was?<br><br>**[Yes=1; No=2; Don't know/Not sure=3]**	A. Heart attack<br>B. Angina<br>C. Heart failure<br>D. Valve disease<br>E. Hole in the heart	☐<br>☐<br>☐<br>☐<br>☐<br>☐<br>☐

	F. Not informed about the nature of the problem<br>G. Others	If "1" for other, please specify ___________ ___________

If "1" for heart attack then fill the following questions otherwise skip to Q3.13	
3.6 If heart attack, how many attacks you have had till now?	☐☐
	**latest event (Heart attack)**
3.7 Date of heart attack	☐☐ ☐☐☐☐<br>Month Year
3.8 What symptoms did you have during this event?<br>A. Chest pain/ discomfort >20 minutes<br>B. Pain radiating to arm, shoulder or neck<br>C. Sweating or vomiting<br>D. Others	[Yes=1; No=2; Not sure=3]<br>☐<br>☐<br>☐<br>☐<br>If others please specify ______________________

3.9 How long these symptoms were present before you met doctor?	☐☐ ☐☐ ☐☐<br>Weeks Days Hours
3.10 Were you hospitalized for this event?<br>**[Yes=1; No=2]**	☐
	**If yes, go to Q3.11 otherwise skip to Q3.12**
3.11 If hospitalized for this event, what procedure did they do in the hospital?<br>**(yes=1, no=2, don't know=3)**	

1) Angioplasty (Stent)<br>2) Coronary Artery bypass surgery (Bypass)<br>3) Thrombolytic therapy<br>4) Only medicines<br>5) Others	☐<br>☐<br>☐<br>☐<br>☐<br>If others please specify<br>_____________________
	**If Q3.11 is filled then skip to Q3.13**
3.12 If **not** hospitalized for this event, where did you take treatment?<br>**(yes=1, no=2, don't know=3)**<br>a) Visited allopathic doctor and took treatment as outpatient<br>b) Visited Ayurveda/homeopathic other traditional healers<br>c) Others	☐<br>☐<br>☐ If others, please specify_____________________

3.13 Are you taking any treatment for heart disease currently?<br>**[Yes=1; No=2]**	A. Allopathic drugs (English /modern)<br>B. Traditional medicine (other than Yoga)<br>C. Yoga<br>D. Others	☐<br>☐<br>☐<br>☐<br>If others please specify<br>_____________________
3.14 **For all participants:** Have you ever undergone coronary angioplasty or stent?<br>(This is a procedure to put stent in the heart blood vessels to destroy clots)	Yes 1<br>No 2	☐<br>**If "2" skip to 3A-III**
3.15 If yes, when did you have latest procedure?	☐☐ ☐☐☐☐<br>Months Years	
**3A-III: STROKE (Paralytic attack)**		
3.16 Have you **EVER** been told by a doctor that you have stroke (Paralytic attack)?	☐<br>**If "2" or "3" skip to *3A-IV***	

[Yes=1 ; No=2; *Don't know=3*]	
3.17 If yes, how many times?	**Number of times** ☐☐
	**Latest Stroke**
3.18 Date of the stroke	☐☐ ☐☐☐☐<br>Month Year
3.19 What symptoms did you experience?<br>A. Did you become unconscious or drowsy?<br>B. Was there loss of vision?<br>C. Was there weakness in face or limbs?<br>D. Was there weakness in on limb/half of the body?<br>E. Was there difficulty in speaking?<br>F. Was there disturbances of balance or walking?<br>G. Was there trauma to the head or neck?	**[Yes= 1; No= 2; Not sure/ Don't remember=3]**<br>☐<br>☐<br>☐<br>☐<br>☐<br>☐<br>☐
	**If "2" or "3" in all the boxes skip to Q3.21**
3.20 Was duration of any symptoms > 24 hours?	**[Yes= 1; No= 2; Not sure/ Don't remember=3]**<br>☐
3.21 Who diagnosed the stroke?<br>a) MBBS doctor 1<br>b) Ayurveda/homeopathic/ 2<br>c) traditional healer<br>d) Others 3<br>e) Not sure/ Don't remember 4	☐<br>If others please specify<br>____________________
3.22 Were you hospitalized for this stroke?	**[Yes= 1; No= 2; Not sure/ Don't remember=3]**<br>☐
	**If "2" skip to Q3.24 and if "3" skip to Q3.25**

3.23 If hospitalized for this stroke, was CT scan or MRI done?	**[Yes= 1; No= 2; Not sure/ Don't remember=3]** ☐	
3.24 If not hospitalized, why?<br><br>a) Visited allopathic doctor and<br>b) took treatment as outpatient 1<br><br>c) Visited Ayurveda/homeopathic<br>d) /other traditional healers 2<br><br>e) Others 3<br><br>f) Not sure/ Don't remember 4	☐<br><br>If others please specify<br><br>_____________________	
3.25 Do you have a residual disability in any part of the body?	Yes 1<br>No 2	☐<br>**If "2" skip to Q3.27**
3.26 If **'YES'**, does it involve the following?<br><br>***[Yes=1; No=2]***	A. Paralysis of leg/foot<br>B. Paralysis of arm/hand<br>C. Weakness of leg/foot<br>D. Weakness of arm/hand<br>E. Defect of speech<br>F. Defect of vision<br>G. Urinary incontinence<br>H. Any other weakness	☐<br>☐<br>☐<br>☐<br>☐<br>☐<br>☐<br>☐<br><br>If other, please specify<br><br>____________________
3.27 Are you **advised** to continue any medication after your paralytic attack?	Yes 1<br>No 2	☐
**3A-IV: LIVER DISEASE**		
3.28 Have you EVER been told by a doctor that you have jaundice?	Yes 1<br>No 2<br>Don't know 3	☐<br>**If "2" or "3"skip to section 4**
3.29 If yes, when was it diagnosed?<br><br>**"write the date of latest event (jaundice)**	☐☐ ☐☐☐☐<br>**MM YYYY**	
**Section:-4 DRUG AND FOOD SUPPLEMENT INFORMATION**		
4.1 In the past one week, have you taken any Allopathic drug (English / modern) for a disease or any food supplements ?	**Yes= 1; No =2**	☐

		**If “2” go to “Section 5”**

4.2 If yes, provide details of all the medication/food supplement that the participant is taking at the time of survey in the below columns

**Name of the drug/food supplement and its contents (Write in CAPTIAL letters)**	Dose of the drug	**Since when are you taking this drug/ food supplement?**	
			**Select the appropriate time measure**<br><br>**[Years=1, Months=2, Week=3, Days=4]**
a)		☐☐	☐
b)		☐☐	☐
c)		☐☐	☐
d)		☐☐	☐
e)		☐☐	☐
f)		☐☐	☐
g)		☐☐	☐
h)		☐☐	☐
i)		☐☐	☐
j)		☐☐	☐

SECTION-5: FAMILY HISTORY

5.1 Has anyone of these in your family suffered from any of the following diseases, before the age of 60 years? (eg: father, mother, son, daughter, brother sister)<br><br>***[Yes=1; No=2; Don’t know=3]***	a) Hypertension (High Blood Pressure)<br>b) Heart disease*<br>c) Diabetes mellitus (High Blood Sugar)<br>d) Stroke (Paralytic Attack)<br>e) Cancer<br>f) Liver disease<br><br>***Angina/ heart attack/heart failure**	☐<br>☐<br>☐<br>☐<br>☐<br>☐

SECTION-6: QUALITY OF LIFE		
**Under each heading, please mention the number that describes your health today**		
6.1 Mobility	I have no problems in walking about 1<br>I have slight problems in walking about 2<br>I have moderate problems in walking about 3<br>I have severe problems in walking about 4<br>I am unable to walk about 5	☐
6.2 Self- Care	I have no problems in bathing or dressing myself 1<br>I have slight problems in bathing or dressing myself 2<br>I have moderate problems in bathing or dressing myself 3<br>I have severe problems in bathing or dressing myself 4<br>I am unable to bath or dress myself 5	☐
6.3 Usual Activities (e.g.. work,study housework family or leisure actvities)	I have no problems doing my usual activities 1<br>I have slight problems doing my usual activities 2<br>I have moderate problems doing my usual activities 3<br>I have severe peoblems doing my usual activities 4<br>I am unable to do my usual activites 5	☐
6.4 Pain/ Discomfort	I have no pain or discomfort 1<br>I have slight pain or discomfort 2<br>I have moderate pain or discomfort 3<br>I have severe pain or discomfort 4<br>I have extreme pain or discomfort 5	☐
6.5 Anxiety/ Depression	I am not anxious or depressed 1<br>I am slightly anxious or depressed 2<br>I am moderately anxious or depressed 3<br>I am severely anxious or depressed 4<br>I am extremely anxious or depressed 5	☐

- We would like to know how good or bad your health is TODAY.
- This scale is numbered from 0 to 100.
- 100 means the best health you can imagine.
  0 means the worst health you can imagine.
- Mark an X on the scale to indicate how your health is TODAY.
- Now, please write the number you marked on the scale in the box below.

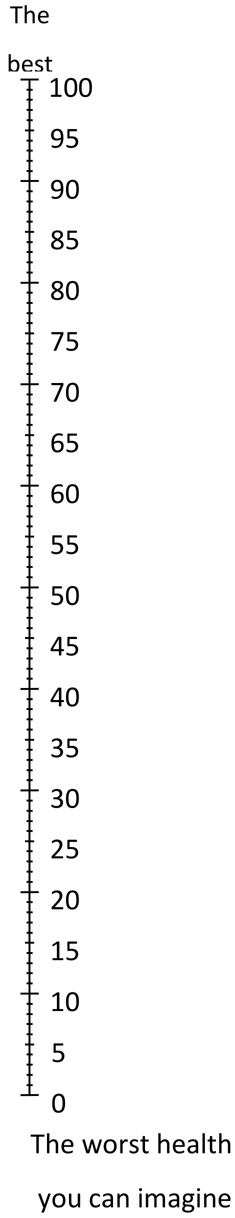

Supplement: S1 Questionnaire — (PDF) [file pone.0263768.s003.pdf]
