# Supplementary material for: Rural-Urban differentials in prevalence, spectrum and determinants of Non-alcoholic Fatty Liver Disease in North Indian population
Source: PLoS One. 2022 Feb 10;17(2):e0263768. doi: 10.1371/journal.pone.0263768 (PMC8830644; doi:10.1371/journal.pone.0263768)
Supplement: Attachment — Submitted filename: Rural-Urban differentials in prevalence, spectrum and determinants of Non- alcoholic Fatty Liver Disease in North Indian population.docx [file pone.0263768.s004.docx]

This cross-sectional study reports the prevalence of NAFLD and analyzes the associated risk factors in appropriate measure. Several limitations were disclosed sufficiently, such as the representative of study population.

1. Why do author select "aged 30-60 years" to instead of "aged 20-80 years" or "aged 20-60 years"? How is different "aged 20-30 years" ?

2. Are all patients with Diabetes type2?

3. Why don't you record the data about alcohol and autoimmune disease?
