# Supplementary material for: Rural-Urban differentials in prevalence, spectrum and determinants of Non-alcoholic Fatty Liver Disease in North Indian population
Source: PLoS One. 2022 Feb 10;17(2):e0263768. doi: 10.1371/journal.pone.0263768 (PMC8830644; doi:10.1371/journal.pone.0263768)
Supplement: Attachment — Submitted filename: Response to Reviewers.docx [file pone.0263768.s005.docx]

Daisuke Tokuhara	Date: 10^th^ January 2022
Academic Editor
PLOS ONE

**Subject:** PLOS ONE Decision: Revision required [PONE-D-21-12623] - [EMID:7047b08d4ca3e067]

Dear Dr Tokuhara,

We thank the reviewers and editors for the thoughtful and valuable comments to improve the manuscript. We are now submitting the revised manuscript incorporating the changes suggested in the comments. Below is our point-by-point response to the editor and reviewers’ comments. We have also attached the revised manuscript- both clean and track-changes versions.

We hope the revised version of manuscript is suitable for publication.

We would be happy to provide further clarifications if required.

Sincerely,

Dr. Nikhil Tandon.

| **S. No.** | **Comments to the Author** | Response |
| --- | --- | --- |
| **Editor’s comments** | | |
| 1 | Please ensure that your manuscript meets PLOS ONE's style requirements, including those for file naming. The PLOS ONE style templates can be found at  https://journals.plos.org/plosone/s/file?id=wjVg/PLOSOne_formatting_sample_main_body.pdf and  https://journals.plos.org/plosone/s/file?id=ba62/PLOSOne_formatting_sample_title_authors_affiliations.pdf | We have formatted the revised manuscript as per the journal’s requirement.  All major Level 1 headings- ‘Abstract in line no. 28, Introduction in line no. 53, Participants and Methods in line no. 93, Result in line no. 175, Discussion in line no 278, Acknowledgments in line no 385, References in line no. 399, Supporting information in line no. 571’ have been modified as bold with font size of 18pt in the ‘manuscript’.  The level 2 heading of subsection- ‘abstract sub-headings, study population and design in line no 94, sampling strategy in line no 105, data collection in line no. 122, statistical analysis in line no 157’ have been made bold with font size of 16pt.  The font size of text has been kept standard (12pt) size with double spacing.  We have replaced the word ‘supplementary Table 1’ and ‘Supplementary Table 2’ with word ‘S1 Table’ and ‘S2 Table’ respectively in line no. 219 and 275 in manuscript. The names of the supplementary tables have also been changed accordingly as S1 Table and S2 Table respectively and have been uploaded as a separate file “S1 and S2 Table” with manuscript.  We have deleted the word ‘The’ in line no. 57. The word ‘were’ has been added in line no. 81. The word ‘full’ has been replaced by ‘entire’ in line no.87. The word ‘studies’ has been replaced by ‘rural and urban components of the study’ in line no. 105. The word ‘The’ has been replaced by ‘A’ in line no. 133. The word ‘Homa IR” has been replaced by HOMA-IR in table 4 and in line no. 200. The sentence ‘only one radiology expert read all the ultrasound scans’ has been paraphrased as ‘All the ultrasound scan were read by a single radiology expert’ in line no. 348. The word ‘the’ has been added in line no. 349. The sentence in line no. 370 has been paraphrased as ‘A probable reason for higher refusal in rural areas could be the travel distance between the villages and the nearest city where the evaluation (including USG and VCTE) was being performed.’ The word ‘heartly’ has been replaced by ‘sincerely’ in line no. 393.  The word ‘Figure’ has been replaced by ‘Fig’ in line no. 177 and 264 in manuscript. The Figure legend has been added in the manuscript as ‘Fig 1. Flow chart showing recruitment of the participants in the study’ and Fig 2. Prevalence of NAFLD on ultrasound by age and sex among urban and rural study subjects in line no 182 and 266 respectively. The ‘Fig 1’ and ‘Fig 2’ have been saved in ‘tiff’ format and have been uploaded as a separate file.  A heading of ‘Supporting Information’ has been added at the end of manuscript at line no. 571 where name of all supporting information files (S1 Table, S2 Table and S1 NAFLD Questionnaire) have been listed. |
| 2 | Please review your reference list to ensure that it is complete and correct. If you have cited papers that have been retracted, please include the rationale for doing so in the manuscript text, or remove these references and replace them with relevant current references. Any changes to the reference list should be mentioned in the rebuttal letter that accompanies your revised manuscript. If you need to cite a retracted article, indicate the article’s retracted status in the References list and also include a citation and full reference for the retraction notice. | We have verified all the cited references. We found none of the cited articles have been retracted. Please let us know if there is any question or concern about a particular reference cited by us. |
| 3 | Please include additional information regarding the survey or questionnaire used in the study and ensure that you have provided sufficient details that others could replicate the analyses. For instance, if you developed a questionnaire as part of this study and it is not under a copyright more restrictive than CC-BY, please include a copy, in both the original language and English, as Supporting Information. | Yes, we are happy to provide the questionnaire used in this study. We have uploaded the questionnaire as supporting file ‘S1 NAFLD Questionnaire. NAFLD-Annexures1’ separately in the submission.  We have also listed ‘S1 NAFLD Questionnaire. NAFLD-Annexures1’ under heading of ‘Supporting Information’ in the manuscript in line no 576. |
| 4 | Please provide additional details regarding participant consent. In the ethics statement in the Methods and online submission information, please ensure that you have specified whether consent was informed. | We have added a statement stating the participant's consent in the manuscript in line no. 115 |
| 5 | Please ensure that you include a title page within your main document. We do appreciate that you have a title page document uploaded as a separate file, however, as per our author guidelines (<http://journals.plos.org/plosone/s/submission-guidelines#loc-title-page>) we do require this to be part of the manuscript file itself and not uploaded separately. Could you therefore please include the title page into the beginning of your manuscript file itself, listing all authors and affiliations. | We have placed the title page as per recommendation. |
| 6 | In your Data Availability statement, you have not specified where the minimal data set underlying the results described in your manuscript can be found. PLOS defines a study's minimal data set as the underlying data used to reach the conclusions drawn in the manuscript and any additional data required to replicate the reported study findings in their entirety. All PLOS journals require that the minimal data set be made fully available. For more information about our data policy, please see http://journals.plos.org/plosone/s/data-availability.  Upon re-submitting your revised manuscript, please upload your study’s minimal underlying data set as either Supporting Information files or to a stable, public repository and include the relevant URLs, DOIs, or accession numbers within your revised cover letter. For a list of acceptable repositories, please see http://journals.plos.org/plosone/s/data-availability#loc-recommended-repositories. Any potentially identifying patient information must be fully anonymized.  Important: If there are ethical or legal restrictions to sharing your data publicly, please explain these restrictions in detail. Please see our guidelines for more information on what we consider unacceptable restrictions to publicly sharing data: http://journals.plos.org/plosone/s/data-availability#loc-unacceptable-data-access-restrictions. Note that it is not acceptable for the authors to be the sole named individuals responsible for ensuring data access.  We will update your Data Availability statement to reflect the information you provide in your cover letter. | The data can’t be shared publicly because the consent for data sharing was not obtained from study participants. The data set used in this study are available at the Research Section of the All India Institute of Medical Sciences, New Delhi. The researchers meeting the criteria can access the data by writing an email to “[deanresearchaiims@gmail.com](mailto:deanresearchaiims@gmail.com)” with the subject line "Request for data for the manuscript Rural-Urban differentials in prevalence, spectrum and determinants of Non-alcoholic Fatty Liver Disease in North Indian population (Author: Tandon)”. |
| 7 | Your ethics statement should only appear in the Methods section of your manuscript. If your ethics statement is written in any section besides the Methods, please move it to the Methods section and delete it from any other section. Please ensure that your ethics statement is included in your manuscript, as the ethics statement entered into the online submission form will not be published alongside your manuscript. | We have moved ethics statement from Introduction section to Participants and Methods of manuscript in line no. 105 as per suggestion. |
| **Reviewer 1 comment** | | |
| 1 | Why do author select "aged 30-60 years" to instead of "aged 20-80 years" or "aged 20-60 years"? How is different "aged 20-30 years" ? | This study was conducted among pre-existing cohort participants of ongoing CARRS cohort study in urban areas and earlier ICMR funded coronary heart disease participants in rural areas. The choice of lower age was constrained by this and not a conscious decision. The CARRS cohort was initiated in 2010 recruiting participating > 20 years of age; and given the timed elapsed between that study and the initiation of the present study most participants were around 30 years or older in age. Similarly, the rural component was leveraging a previous study in participants greater than 30 years of age. Hence the lower age limit for eligibility was 30 years.  We restricted the upper age to 60 years, so as to get prevalence of NAFLD in the productive adult population. |
| 2 | Are all patients with Diabetes type2? | While we cannot be sure that all diabetes patients are type 2, in an adult population these are highly likely to be type 2. However, we have only used the term ‘diabetes’ in the manuscript rather than specifying a Type 2 Diabetes Mellitus or Type 1 Diabetes Mellitus. |
| 3 | Why don't you record the data about alcohol and autoimmune disease? | The research aims to study non-alcoholic fatty liver disease (NAFLD) which by definition excludes individuals consuming alcohol>20 gms a day. (ref-4)  We agree that autoimmune disorders are also an important cause of fatty liver, though the overall prevalence is very less. We excluded those participants who had self-reported pre-existing liver disorders. However, it is hard to determine the cause of these pre-existing liver disorders in LMICs like India, where the documentation of causes are not always available. |
| **Reviewer 2 comment** | | |
| 1 | Authors should discuss CAP cut off value in the section of Discussion. CAP is the important tool in this study for the screening of NAFLD. Authors used the cut off value (275dB/m) of CAP for determining the presence of NAFLD based on the previous study (Chalmers J et al. BMJ Open 2019). However there are currently no internationally established cut-off value of CAP of NAFLD. Numerous studies provided or used original cut off or reference value of CAP for NAFLD and healthy individuals in adult, children and adolescents. Results regarding the prevalence of NAFLD may be different according to the used cut off value of the CAP value. Therefore I strongly recommend to cite at least the following references from different countries in the revised manuscript. Then authors should add the study limitation in terms of the use of the cut off or reference value of CAP. For example, discrepancies of the cut off and/or reference value may relate to differences in the study design and populations including disease aetiologies, the prevalence of obesity and extent of subcutaneous adiposity, and the severity of steatosis, which may influence CAP performance.  (Reference 1) Sasso M, et al. Ultrasound Med Biol. 2010;36(11):1825-35. (Reference 2) de Lédinghen V, et al. Liver Int. 2012;32(6):911-8. (Reference 3) Tokuhara D, et al. Plos ONE. 2016;11:e0166683 (Reference 4) Isoura Y, et al. Obes Res Clin Pract. 2020;14(5):473-478 (Reference 5) Chon YE, et al. Liver Int. 2014;34(1):102-9 | We thank the reviewer for the great suggestion. We have now included the suggested references and discussion in the manuscript in line no. 330 as ‘CAP values for the presence of steatosis vary from 215-288 dB/m across studies due to differences in demographics, and laboratory parameters of included patients.’ |
| 2 | Another limitation is that authors have assessed liver fibrosis by means of liver stiffness alone. There are several useful blood-biochemical fibrosis marker or indexes (e.g., hyaluronic acid, type-IV collagen, AST to platelet ratio index [APRI] or FIB-4 index) which are known to be useful in the screening of NAFLD. So, Authors are encouraged to use at least one of those blood-biochemical parameters to assess NASH in the revised manuscript. If there is no serum samples to be examined, authors should discuss the above points in the section of Discussion as the study limitation. | We would like to thank the reviewer for this excellent suggestion, with which we agree. We assessed hepatic fibrosis by liver stiffness measurement values only. Unfortunately, we did not collect data of platelet count to determine the FIB-4 and APRI scores. We have mentioned this as a limitation in the discussion part of the revised manuscript. (Line no. 365). CAP values are affected by multiple variables, including body mass index, etiology of the liver disease, the extent of subcutaneous fat, and severity of hepatic steatosis. The prevalence of NAFLD would vary depending upon the CAP cut-off used for the grading of hepatic steatosis. We used the CAP cut-off published from our centre. The non-availability of the biochemical parameter (platelet count) is another limitation because APRI and FIB-4 index could not be calculated. Therefore, we assessed liver stiffness based on LSM alone. |
| 3 | Generally male population are prone to develop NAFLD rather than female population. Some studies suggest that, in elderly population, female are prone to develop NAFLD because NAFLD-protective role of estrogen are impaired by the menopause. In the current study, prevalence of NAFLD is predominant in female rather than male population especially in the elderly. Please discuss any hypothesis in terms of the sex differences in the current result more deeply.  . | There were no significant differences between men and women in our study, both in univariate and multi-variate analysis. In fact, after adjustment, the risk was lower in women (not significant). As the reviewers say, this might be due to age of the participants. With mean age as 45 years, we expect the protection by estrogen may be lower in this cohort. |
|  | Recently NAFLD and NASH are known to be strongly associated with the gut microbiota. Is there any possibilities that rural and urban differences in prevalence of NAFLD is arised from gut microbiota differencies ? The latest review article (Tokuhara D. Frontiers in Nutrition. 2021;8:700058) well described the role of NAFLD/NASH. It is recommended to shortly discuss the potential involvement of gut microbiome in terms of the results in this study by citing the previous paper | Thank you for the suggestion. We have added the possible explanation of gut microbial and NAFLD and urban and rural differences in the discussion section of the revised manuscript as ‘Additionally, other factors might explain the urban-rural differences, such as gut microbiota. The role of gut microbiota in the pathogenesis of NAFLD is still evolving. Changes in gut-liver homeostases, such as breakdown of the gut barrier, portal transfer of bacterial endotoxin (lipopolysaccharide) to the liver, changed bile acid profiles, and lower concentrations of short-chain fatty acids, all contribute to the development of NAFLD’. |
